# Synthesis by extrusion: continuous, large-scale preparation of MOFs using little or no solvent†
†Electronic supplementary information (ESI) available. See DOI: 10.1039/c4sc03217a



**DOI:** 10.1039/c4sc03217a

**Published:** 2015-01-08

**Authors:** Deborah Crawford, José Casaban, Robert Haydon, Nicola Giri, Tony McNally, Stuart L. James

**Affiliations:** a School of Chemistry and Chemical Engineering , Queen's University Belfast , David Keir Building, Stranmillis Road , Belfast , BT9 5AG , UK . Email: s.james@qub.ac.uk; b MOF Technologies Ltd , 63 University Road , Belfast BT7 1NF , UK . Email: contact@moftechnologies.com; c IINM , WMG , University of Warwick , Coventry , CV4 7AL , UK . Email: t.mcnally@warwick.ac.uk

## Abstract

Grinding solid reagents under solvent-free or low-solvent conditions (mechanochemistry) is emerging as a general synthetic technique which is an alternative to conventional solvent-intensive methods. However, it is essential to find ways to scale-up this type of synthesis if its promise of cleaner manufacturing is to be realised. Here, we demonstrate the use of twin screw and single screw extruders for the continuous synthesis of various metal complexes, including Ni(salen), Ni(NCS)_2_(PPh_3_)_2_ as well as the commercially important metal organic frameworks (MOFs) Cu_3_(BTC)_2_ (HKUST-1), Zn(2-methylimidazolate)_2_ (ZIF-8, MAF-4) and Al(fumarate)(OH). Notably, Al(fumarate)(OH) has not previously been synthesised mechanochemically. Quantitative conversions occur to give products at kg h^–1^ rates which, after activation, exhibit surface areas and pore volumes equivalent to those of materials produced by conventional solvent-based methods. Some reactions can be performed either under completely solvent-free conditions whereas others require the addition of small amounts of solvent (typically 3–4 mol equivalents). Continuous neat melt phase synthesis is also successfully demonstrated by both twin screw and single screw extrusion for ZIF-8. The latter technique provided ZIF-8 at 4 kg h^–1^. The space time yields (STYs) for these methods of up to 144 × 10^3^ kg per m^3^ per day are orders of magnitude greater than STYs for other methods of making MOFs. Extrusion methods clearly enable scaling of mechanochemical and melt phase synthesis under solvent-free or low-solvent conditions, and may also be applied in synthesis more generally.

## Abstract 2

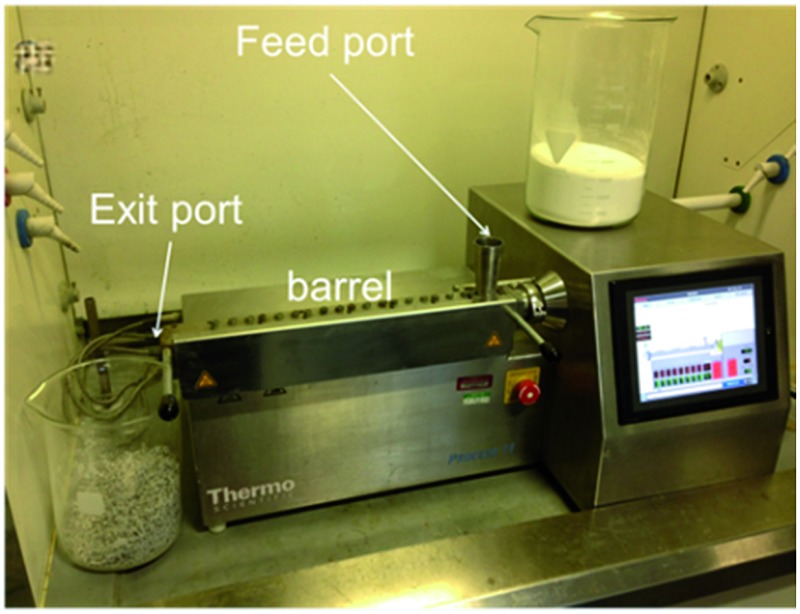
Continuous flow mechanochemical and melt-phase synthesis at kg h^–1^ rates from solid reagents and either no solvent, or only minimal solvent, is reported.

## 

Demonstrating the scalability of solvent-free chemical synthesis is critical in moving towards more sustainable chemical processes. Mechanochemical synthesis, which typically involves solid reagents being ground together with either no solvent or only minimal solvent, and has long been used in the context of insoluble extended inorganic materials, is now emerging as a generally effective method for molecular synthesis.^1^ However, most reports of molecular mechanochemical synthesis normally describe the use of ball mills for reactions at less than one gram scale.^1*b*^ Recently, progress has been made in scaling up molecular mechanochemical synthesis by milling, typically to some tens or occasionally hundreds of grams, by using planetary ball mills, attrition mills or stirred media mills,^2a–d^ and although 50 kg scale mechanochemical synthesis of drug carrier composites has been reported to be successful by ball milling,^2*e*^ the generality of ball milling for mechanochemical synthesis at large (multi-kg) scale has yet to be demonstrated.

The term extrusion refers to a family of continuous processing techniques in which materials are forced through constrained spaces.^3^ An example is twin screw extrusion (TSE) in which two co- or counter-rotating screws transport material along a barrel (in an Archimedean fashion) and subject it to shearing and compression forces due to the presence of mixing/kneading elements incorporated into the screw design. Extrusion is widely done on industrial scales, such as in blending polymers.^3^ Reactive extrusion is also known in which polymer functional groups undergo a chemical reaction during the process.^3*b*^ Recently, TSE has been demonstrated for the formation of organic co-crystals,^4^ in particular for ibuprofen–nicotinamide,^4*a*^ caffeine–oxalic acid^4*b*^ and AMG-517-sorbic acid^4b,c^ co-crystals under melt^4*a*^ or non-melt^4*b*^ conditions. However, it is still questionable whether extrusion methods can be applied to covalent chemical synthesis. For example, the breaking and formation of covalent bonds involves much greater energy than do the supramolecular interactions involved in the formation of co-crystals, and the kinetics of such reactions are often much slower (residence times in TSE are often only a few minutes or even seconds depending on the processing parameters applied). Also, the considerable mixing, shearing and compression forces which can occur in TSE could render any products amorphous, which may not be desirable. Further practical challenges include inducing equivalent transport rates of solid reactants with diverse particle sizes and shapes so that correct stoichiometry is maintained along the barrel, in addition to the changing rheological and mechanical properties of the reaction mixture as the reaction progresses.

We report here that, surprisingly, TSE can indeed be remarkably effective for the synthesis of a range of covalently bonded metal–organic compounds, with either no added solvent or only minimal added solvent, at kg h^–1^ rates (and potentially much greater rates) to give products in high yield, purity and crystallinity.

In the current work, a ThermoFisher Process-11 Twin Screw Extruder was used (Fig. 1 and ESI†) with a screw configuration consisting of a sequence of alternating conveying and kneading zones.

**Fig. 1:**
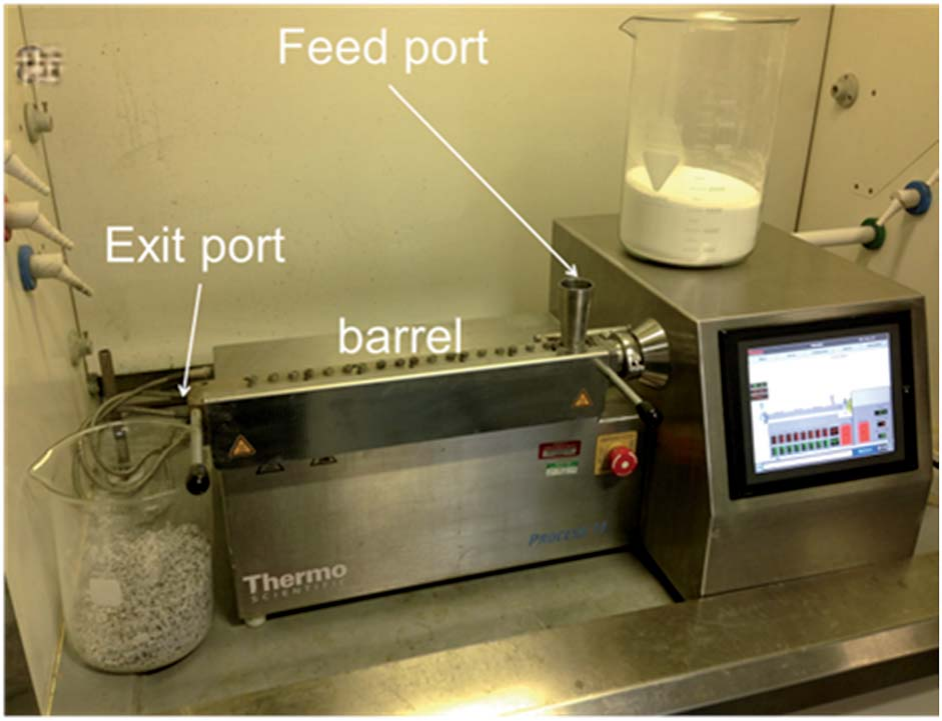
Twin screw extruder with key parts highlighted. The two screws which convey and knead the reactants are housed in the barrel.

We initially investigated relatively simple nickel(ii) complexation reactions, one of which has recently been reported to occur under ball milling conditions,^5^ at modest scales. Solid reactants (15–20 g) were mixed by hand for 30 seconds before being fed into the extruder. Using a screw speed of 55 rpm and a feed rate of 1–1.5 g min^–1^, a mixture of green Ni(OAc)_2_·4H_2_O and the yellow tetradentate chelating ligand H_2_salen reacted to give Ni(salen) which was extruded as a yellow-brown powder (Fig. 2, see ESI† for details). To achieve complete conversion under these conditions, a small amount of solvent, methanol (3.4 mol equiv., approximately 0.45 ml in 19 g of reactants), needed to be added to the mixture of reactants before extrusion. Thus, small amounts of solvent can have a similar enabling effect in synthesis by extrusion as it does in ball mills.^6^ PXRD showed that the product was highly crystalline, and more specifically that it had adopted its dimeric solid form^7*a*^ in which the Ni centres are bridged by phenolic O atoms. Interestingly, this result contrasts with the recently reported ball milling synthesis of Ni(salen) starting from the same reactants, also in the presence of a small amount of methanol, which gave the product in an alternative, previously unknown polymorph.^5^ This demonstrates that TSE may give alternative polymorphs to those obtained by ball milling due to the contrasting reaction and crystallisation conditions. In a second reaction, a mixture of olive-green Ni(NCS)_2_ and white PPh_3_ together with a small amount of methanol (3.4 mol equiv., approximately 0.57 ml in 20 g of reactants) reacted quantitatively under similar extrusion conditions to give *trans*-Ni(NCS)_2_(PPh_3_)_2_ which was extruded as a bright orange powder. PXRD (see ESI†) showed in this case that the product was obtained as the single known polymorph.^7*b*^ In both of these Ni(ii) complexation reactions the exact stoichiometric ratios of metal salt to ligand were used (*i.e.* no excess of either reagent was needed), the residence times were, remarkably, less than 2 minutes to achieve quantitative conversions.

**Fig. 2:**
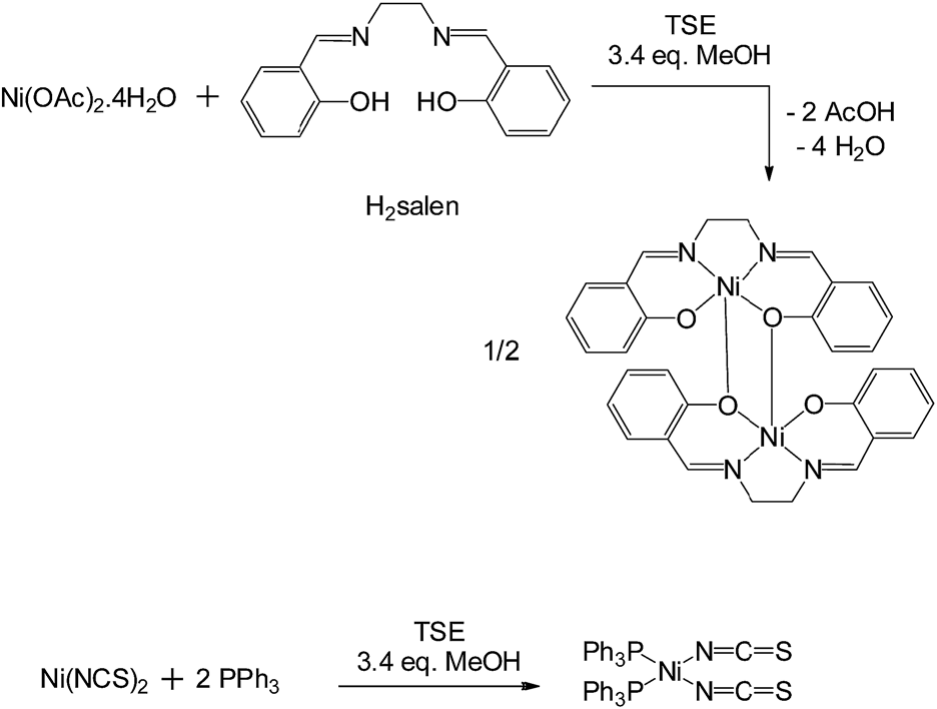
Reaction scheme for the formation of nickel(ii) complexes by twin screw extrusion (TSE).

We then explored the synthesis of MOFs (metal organic frameworks, Fig. 3) at larger scales. Since the mechanochemical synthesis of a porous MOF Cu(INA)_2_ (INA = isonicotinate) was reported in 2006,^8^ a range of MOFs has been prepared by ball milling, but normally at less than 1 g scale.^1c,9^ The synthesis of MOFs is potentially more demanding than that of the above coordination complexes because, in addition to forming the correct product, the functional porosity of the final activated material is critical to the usefulness of any method of synthesis. We found that Cu_3_(BTC)_2_ (copper benzene-1,3,5-tricarboxylate, HKUST-1),^10^ an archetypal MOF, could be prepared from copper(ii) hydroxide and benzene-1,3,5-tricarboxylic acid at the appropriate 3 : 2 mole ratio, by solvent-assisted TSE employing screw speeds ranging from 55–250 rpm and feed rates of 5 g min^–1^, rising to 17 g min^–1^. Water is the only by-product of this reaction. Quantitative reactions under these conditions required the addition of a larger amount of solvent (industrial alcohol, 32 wt%) than in the above examples, but the solvent still constitutes a minor part of the reaction mixture. It is notable that the maximum throughput reached 1 kg h^–1^. Subsequent activation (see ESI†) provided dark blue-purple powders with N_2_ Brunauer–Emmett–Teller (BET) surface areas of 1600–1850 m^2^ g^–1^ depending on the conditions used. These figures compare well with those in the literature for this material prepared by conventional methods.^10^

**Fig. 3:**
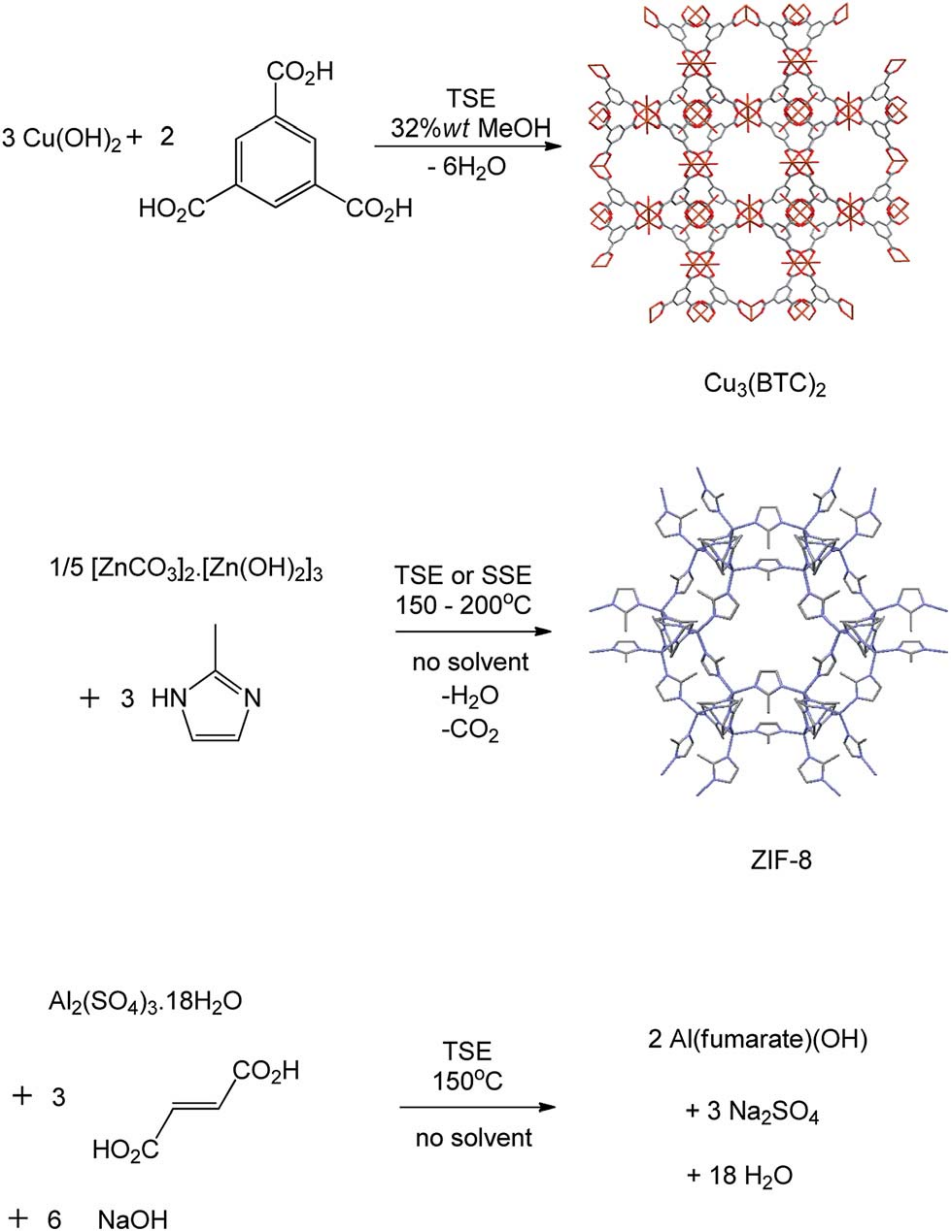
Reaction schemes for synthesis of metal organic frameworks Cu_3_(BTC)_2_ (HKUST-1), Zn(2-methylimidazolate)_2_ (ZIF-8) by twin screw extrusion (TSE) or single screw extrusion (SSE) and Al(fumarate)(OH).

A second archetypal MOF, Zn(2-methylimidazolate)_2_ (ZIF-8),^11^ was also successfully synthesised from basic zinc carbonate, [Zn_2_(CO_2_)_2_]·[Zn_3_(OH)_6_], and 2-methylimidazole (Zn to 2-methylimidazole mole ratio 2 : 3), by solvent-free TSE at screw speeds of 55–95 rpm and feed rates of 5–17 g min^–1^, as shown by PXRD (Fig. 4). Water and CO_2_ are the only by-products in this reaction. To assess scalability of the method, this synthesis was also conducted using a larger, 16 mm, twin screw extruder (Haake Rheomex, see ESI†). An excess of 2-methylimidazole was required for a complete reaction, which is ascribed to the ligand becoming partially occluded in the pores of ZIF-8 as it forms.^12^ Although a solid mixture was fed in without the addition of any solvent, extrusion was conducted most successfully by heating the barrel to 200 °C such that the ligand (m.p. 142–143 °C) would be expected to have melted within the extruder. The ability to control the temperature in the barrel of such extruders represents an advantage over ball milling, since in the latter heating is normally adventitious due to friction and temperature control is not a standard feature. A beige extrudate was collected and activated (see ESI†) to give a white solid with a BET surface area of 1603 m^2^ g^–1^. This value compares well with literature values for this MOF.^11^ As with Cu_3_(BTC)_2_, the throughput rate reached 1 kg h^–1^.

**Fig. 4:**
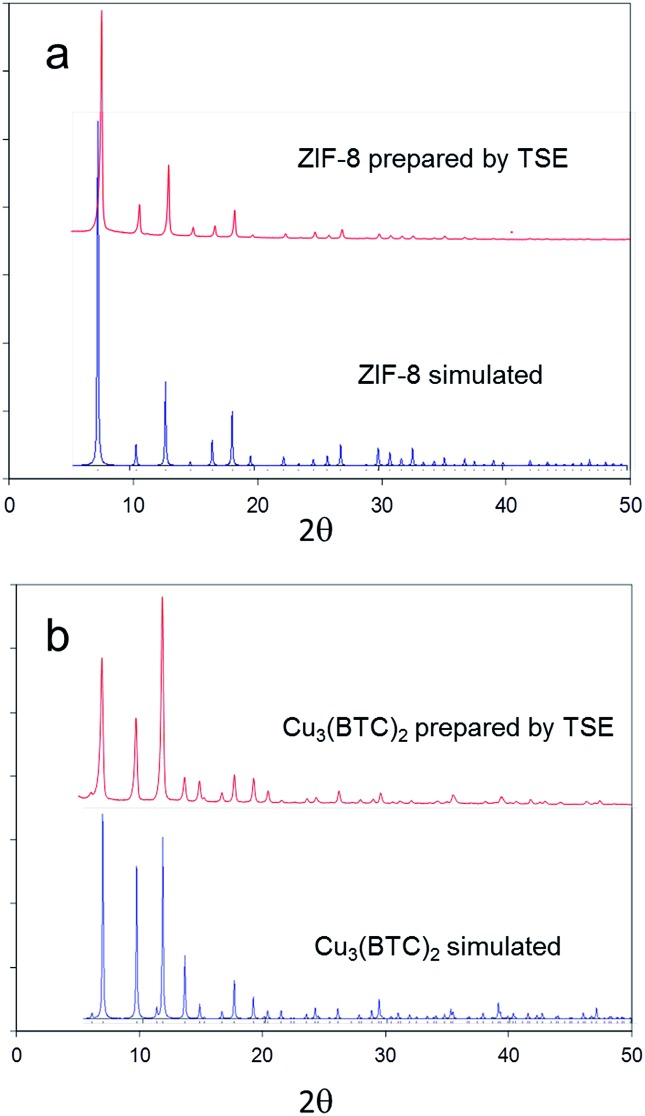
PXRD patterns for ZIF-8 and Cu_3_(BTC)_3_ obtained by twin screw extrusion compared to patterns simulated from single crystal X-ray diffraction data (CSD codes FAWCEN and FIQCEN respectively).

Furthermore, it was found that ZIF-8 could also be prepared by an alternative extrusion method, specifically single screw extrusion (SSE, see ESI†). A screw with a gradually increasing root diameter was employed consisting largely of a conveying section with a short kneading element in the final zone. In SSE work is applied by compression forces generated by a reduction in screw channel depth upon moving from the feeding to the metering section. The process was carried out using a temperature gradient of 30–150 °C along the barrel and a screw speed of 30 rpm. Remarkably, this experimental set up permitted feed rates of up to 70 g min^–1^ (4 kg h^–1^). A beige extrudate was obtained which upon activation gave a white solid with a BET surface area of 1750 m^2^ g^–1^. ZIF-8 (also known as MAF-4) has previously been synthesised by heating neat ZnO and 2-methylimidazole in up to 10 g batches for 12 hours, although it is not clear to what degree such methods involve a melt phase.^11*e*^ Performing synthesis *via* melt phases remains unusual in coordination chemistry^13^ and we are unaware of previous examples of continuous melt phase synthesis in this context.

Finally we explored the synthesis of a third important MOF, Al(fumarate)(OH), which has not previously been synthesised mechanochemically to our knowledge.^14^ As with a range of other MOFs based on Al(iii) and other tri- or tetra-valent metal ions, this material is noted for its very high stability to water, which makes it of potential use in absorption chiller applications,^14*b*^ and, together with its high methane capacity, natural gas storage.^14*a*^ However, relatively few MOFs based on tri- and tetra-valent metal ions have been synthesised mechanochemically to date, exceptions being two series of lanthanide–BTC MOFs.^15^ This may reflect difficulties associated with the generally higher lattice energies of typical metal salt starting materials.^15^ Using the Haake Rheomex 16 mm twin screw extruder at 150 °C, a screw rate of 95 rpm and a feed rate of 10 g min^–1^ (0.6 kg h^–1^) we found that 125 g of a mixture of Al_2_(SO_4_)_3_·18H_2_O, fumaric acid and NaOH in the appropriate stoichiometric ratio readily afforded Al(fumarate)(OH) quantitatively together with the expected Na_2_SO_4_ by-product as shown by PXRD. In this case one pass through the extruder resulted in only partial formation of the product and therefore the mixture was passed through the extruder a total of three times under the same conditions. Washing the product with water removed the Na_2_SO_4_ (see ESI†) to leave Al(fumarate)(OH) with a BET surface area of 1010 m^2^ g^–1^ which is similar to literature values for this material.^14^ Preliminary results suggest that it is also possible to obtain members of the MOF-74 (also known as CPO 27) series (M = Mg, Zn, Co)^16^ by TSE.

The space time yield (STY) is a process parameter which is used as a measure process intensification and generally speaking it should be as high as possible for a profitable process. STYs for the MOF syntheses reported here (taking the reactor volume as the volume of the extruder barrel) are compared with those for solvent-based processes previously reported for the same MOFs^14a,17,18^ in the Table 1. It is remarkable that the values for synthesis by extrusion are between 1 and 3 orders of magnitude greater than those for other methods. These high values result from the absence, or near absence, of solvent which would normally occupy the majority of the volume in a reactor, together with the high reaction rates possible between such highly concentrated (or even neat) reactants under these conditions.

**Table 1:** Comparison of space time yields (STYs) for synthesis of Al(fumarate)(OH), Cu_3_(BTC)_2_ and ZIF-8 by various synthetic methods

MOF	Space time yield (STY)/kg per m^3^ per day	Reference
Al(fumarate)(OH)	>3600	14a
	27 000	This work
Cu_3_(BTC)_2_	494	17a
	225	17b
	144 000	This work
ZIF-8	69.4	17a
	100	17b
	1400	11e
	144 000 (TSE)	This work
	7826 (SSE)	This work

In conclusion, both twin- and single screw extrusion can be effective methods for covalent chemical synthesis under solvent-free, minimal solvent, or melt phase conditions. This study suggests that, subject to a degree of optimisation, synthesis by extrusion can reproduce at large scales in a continuous process what synthesis by ball milling can achieve at small scales in a batch process. It also shows that alternative phases to those obtained by ball milling can in some cases be obtained. It is notable that high throughput rates of several kg h^–1^ were readily achieved and much greater rates of several hundred kg h^–1^ should be possible by using larger scale extrusion equipment which is widely available. Reactions were quantitative and selective, and the quality of the final activated MOFs as judged by BET surface areas and pore volumes was high. It is important also to note that extrusion techniques such as those explored here potentially provide a fascinating and essentially unexplored playing field for the discovery of new synthetic chemistry due to the unusual combination of high compression, shear and mixing forces combined with high temperatures which are readily achieved in this equipment. Clearly, although much work is now needed to gain greater knowledge and understanding of this methodology, these findings open up many possibilities for both the scale-up and the further development of mechanochemical and melt-phase covalent synthesis.
