# Supplementary material for: Synthesis by extrusion: continuous, large-scale preparation of MOFs using little or no solvent
Source: Chem Sci. 2015 Jan 8;6(3):1645–9. doi: 10.1039/c4sc03217a (PMC5639793; doi:10.1039/c4sc03217a)
Supplement: Supplementary file 1 [file SC-006-C4SC03217A-s001.pdf]

Electronic Supplementary Material (ESI) for Chemical Science.
This journal is © The Royal Society of Chemistry 2015

Synthesis by extrusion: Continuous, large-scale preparation of MOFs using little or no solvent

Supplementary information

	page
General aspects	2
Synthesis of Ni(salen)	3
Synthesis of $Ni(NCS)_2(PPh_3)_2$	5
Synthesis of $Cu_3(BTC)_2$	7
Synthesis of $Zn(methylimidazolate)_2$ (ZIF-8)	10
Synthesis of $Zn(methylimidazolate)_2$ (ZIF-8) by SSE	12
Synthesis of Al(fumarate)(OH)	14

General aspects

Extrusion

Twin screw extrusion (TSE) was carried out using either a Thermo Fisher Process 11 Parallel Co-Rotating twin screw extruder, with an l/d ratio of 25, or a Haake Rheomex OS PTW16 co-rotating twin screw extruder with an l/d ratio of 40:1. Screw speeds were varied from 55 - 250 rpm and the temperature from ambient to 200^{0}C. Single screw extrusion (SSE) was conducted with a Dr. Collin E 25M single screw extruder with a l/d ratio of 25 at a screw speed of 30 rpm using a 25 mm diameter PTFE screw of constantly increasing root diameter.

Materials and Measurements

All materials were obtained from Sigma Aldrich UK in >98% purity or Molekula in >96% purity and were used as obtained without further purification. PXRD measurements were carried out on a PANanalytical X'Pert Pro X-ray diffractometer. Copper was used as the X-ray source with a wavelength of 1.5405 Å. All experiments were carried out ex-situ using a spinning stage. Diffractograms were typically obtained from 5–50° with a step size of 0.0167°. Solution state NMR spectra were recorded on a Bruker AM 300 MHz referenced to the residual ^{1}H containing solvent. Chemical shifts (δ) are given in parts per million (ppm) and coupling constants in Hertz (Hz). In situ activation of compounds prior to Brunauer-Emmett-Teller (BET) analysis involved in situ heating at 200^{0}C under vacuum for 2 hours. N_2 adsorption isotherms were obtained at -196^{0}C between relative pressures (p/p^0) of 0.005 to 0.995, using a QuantaChrome Nova 4200e Surface area analyser. Total pore volume was obtained at p/p^0 = 0.995. Elemental analyses were determined by the Analytical Service department of the School of Chemistry and Chemical Engineering (ASEP) using a Perkin-Elmer 2400 CHN microanalyser.

Synthesis of [Ni(salen)]

Nickel(II) acetate tetrahydrate (9.27 g, 0.037 moles), 2,2-[1,2- ethanediylbis[(E)-nitrilomethylidyne]]bis-phenol) (salenH_2, 10g, 0.037 moles) and methanol (HPLC grade, 0.0111, 0.449 mL) were mixed together by hand for 30 seconds. The mixture was fed manually into a ThermoFisher Process 11 Extruder, with a screw speed of 55 rpm (residence time *ca.* 1.5 - 2 minutes). A brown-yellow powder extrudate was obtained whose PXRD pattern was very similar to that simulated from the single crystal X-ray diffraction data for Ni(salen) (Figure 1). A small sample was heated to 80°C for 2h remove any volatiles before solution NMR analysis (Figure 2). NMR data were consistent with literature data.

^{1}H NMR δH (300 MHz, d^{6}-DMSO): 7.88 (s, 2H), 7.23 (d, 2H, J = 6.0 Hz), 7.16 (t, 2H, ^{2}J = 6.5 Hz, ^{3}J = 13.0 Hz), 6.70 (d, 2H, J = 6.0 Hz), 6.50 (t, 2H, ^{2}J = 7.5 Hz, ^{3}J = 15.0 Hz), 3.4 (s, 4H).

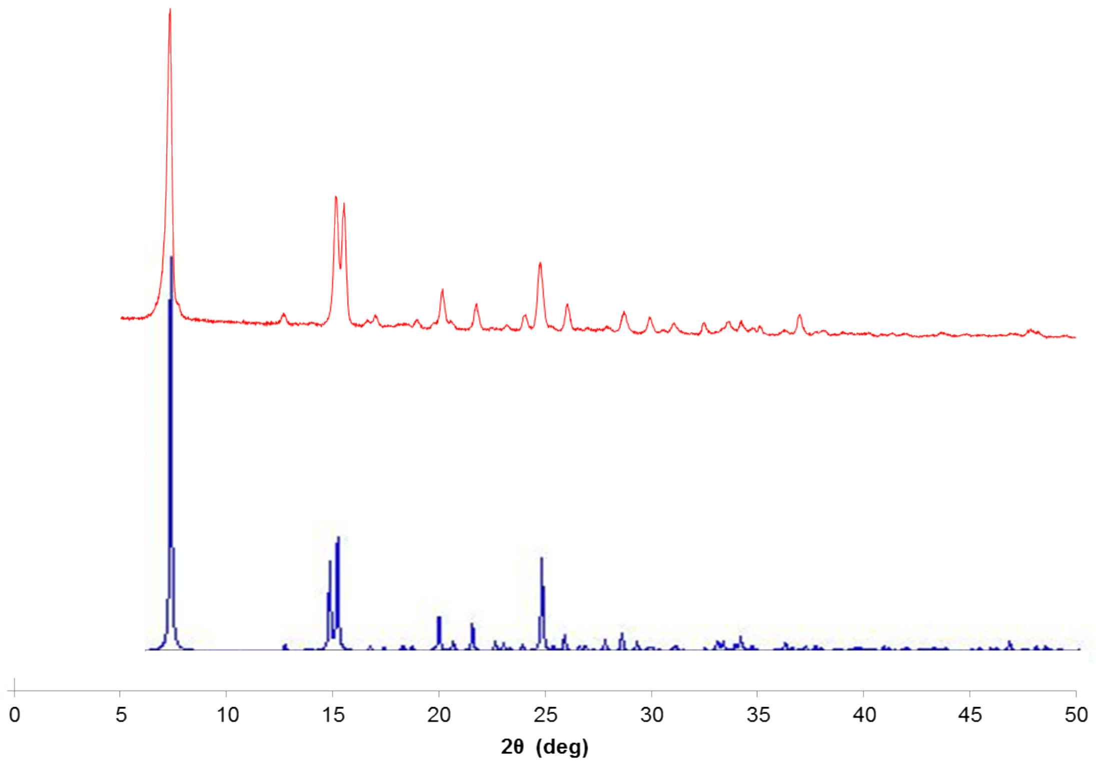

Figure 1 PXRD pattern for Ni(salen) synthesised by TSE (above) and the simulated PXRD patter for Ni(salen) (below, CSD code RITMUD).

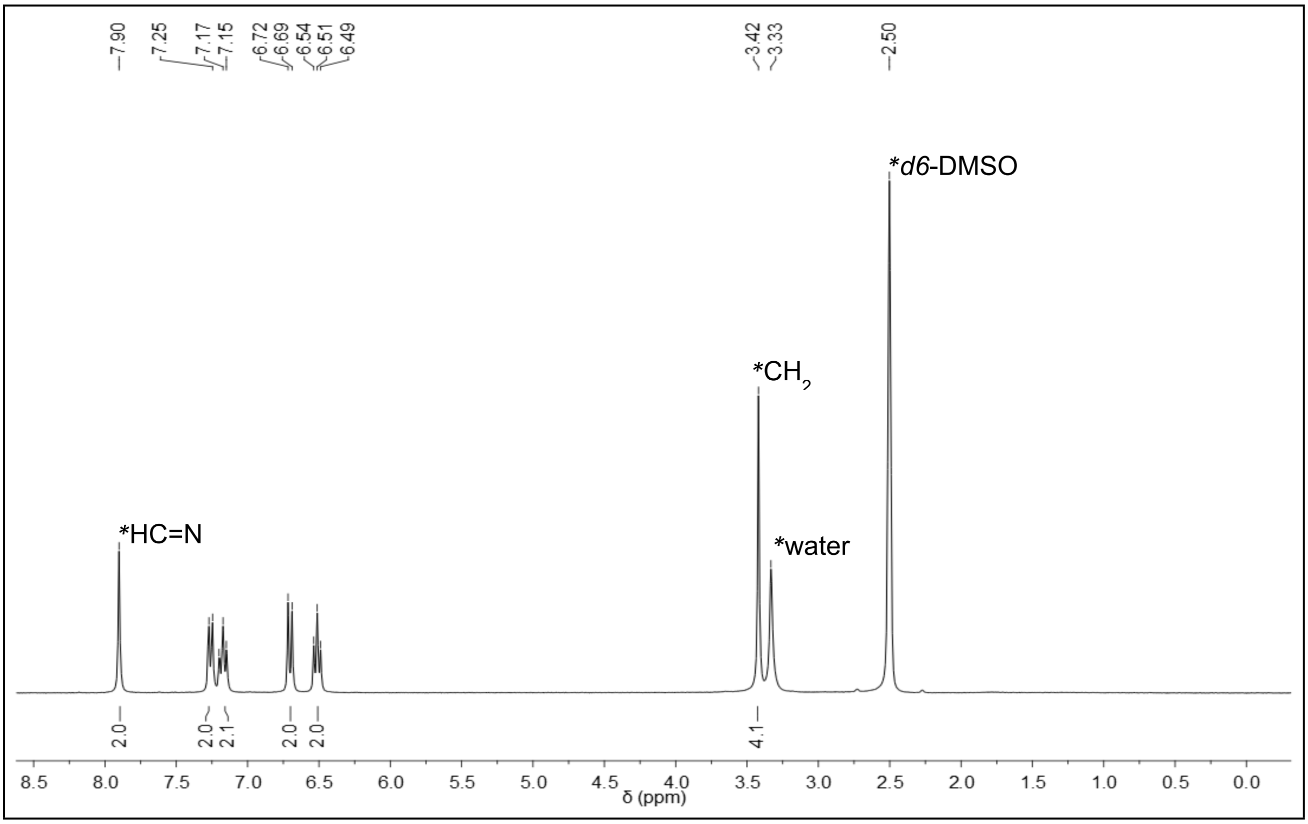

Figure 2 Solution ^1H NMR spectrum (d^6-DMSO) of Ni(salen) synthesised by TSE.

Synthesis of *trans*-[Ni(NCS)$_2$(PPh$_3$)$_2$]

Nickel(II) thiocyanate (5 g, 0.0285 moles), triphenylphosphine (14.98 g, 0.0572 moles) and methanol (HPLC grade, 0.0112 moles, 0.57 mL) were mixed together by hand for 30 seconds. The mixture was fed manually into a ThermoFisher Process 11 Extruder, at a screw speed of 55 rpm (residence time *ca.* 1.5 - 2 minutes) to give an orange powder extrudate whose PXRD pattern was very similar to that of the simulated pattern simulated from the single crystal X-ray diffraction data of *trans*-[Ni(NCS)$_2$(PPh$_3$)$_2$] (Figure 3). A small sample was heated to 80°C for 2h to remove any volatiles before solution NMR analysis. NMR data were consistent with literature data (Figures 4 and 5)..

^{1}H NMR δH (300 MHz, CDCl$_3$): 7.62 - 7.48 (m, 30H). ^{31}P{^{1}H} (300 MHz, CDCl$_3$): 14.6 ppm

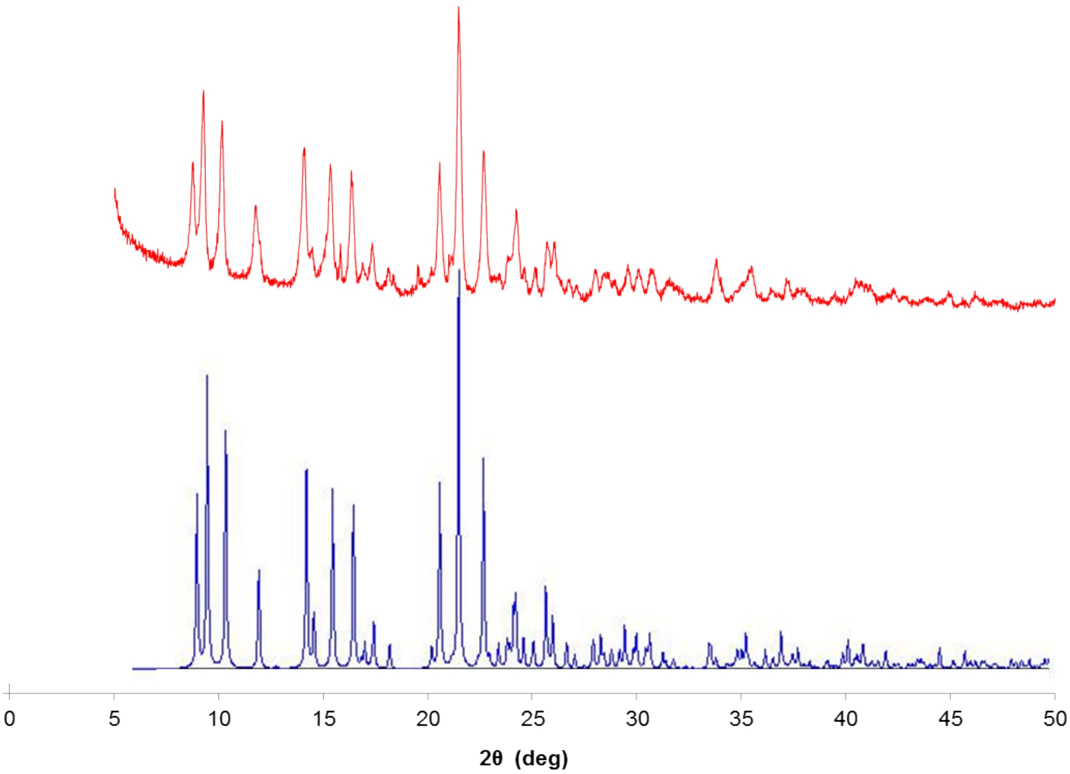

Figure 3 PXRD pattern for *trans*-Ni(NCS)$_2$(PPh$_3$)$_2$ synthesised by TSE (above) and the simulated PXRD pattern for *trans*-Ni(NCS)$_2$(PPh$_3$)$_2$ (below, CSD code GEBZUI).

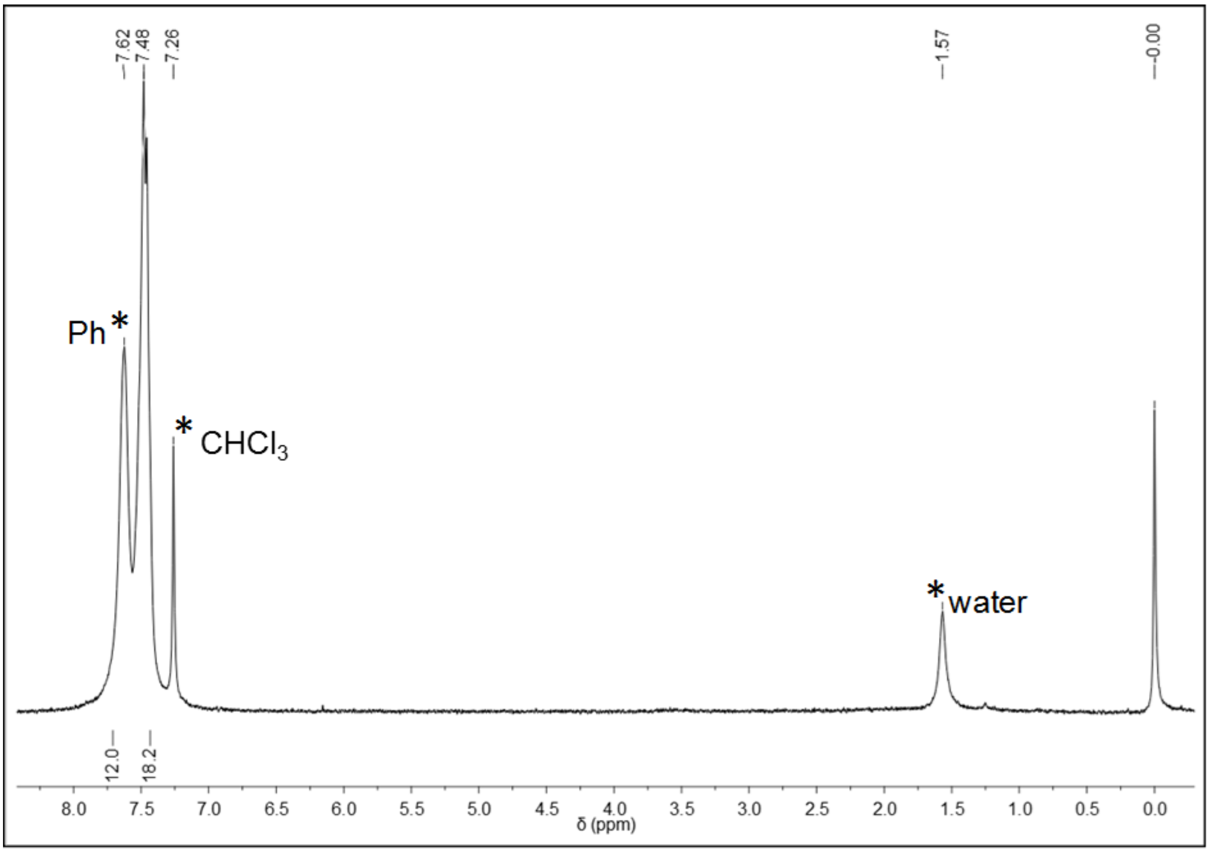

Figure 4 Solution ^{1}H NMR spectrum ($CDCl_3$) for *trans*-$Ni(NCS)_2(PPh_3)_2$ synthesised by TSE.

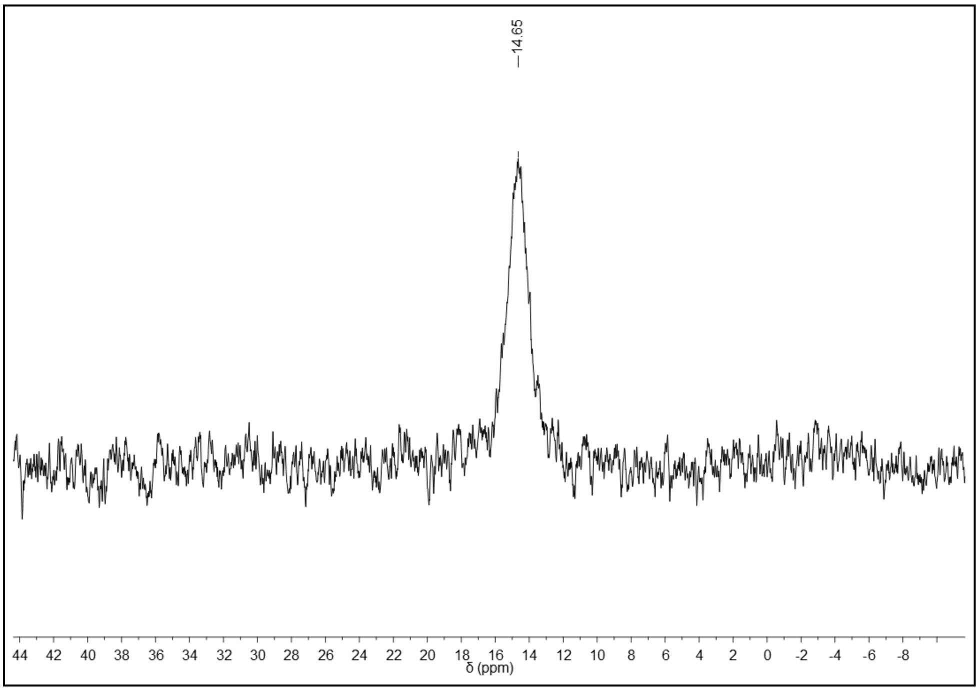

Figure 5 Solution ^{31}P NMR spectrum ($CDCl_3$) for *trans*-$Ni(NCS)_2(PPh_3)_2$ synthesised by TSE.

Synthesis of $Cu_3(BTC)_2$ (HKUST-1)

Copper(II) hydroxide (0.43 moles, 42.0 g), benzene-1, 3, 5-tricarboxylic acid (58.0 g, 0.286 moles) and industrial alcohol (99.9%, 60 ml) were mixed by hand for 30 seconds and fed manually into a ThermoFisher Process 11 at a screw speed of 135 rpm. A light blue powder extrudate was obtained whose PXRD pattern was similar to that simulated from the single crystal data for $Cu_3(BTC)_2$ (Figure 6). 1g of extrudate was stirred for 20 minutes in absolute ethanol (8 ml) and collected by filtration, three times. Oven-drying at 150°C for 2 hours produced a dark purple solid. Similar PXRD patterns were obtained for products formed at screw speeds of 55-250 rpm (Figure 6). N_2 BET Surface area: 1738 m^2g^{-1}; pore volume: 0.834 cm^3g^{-1}.

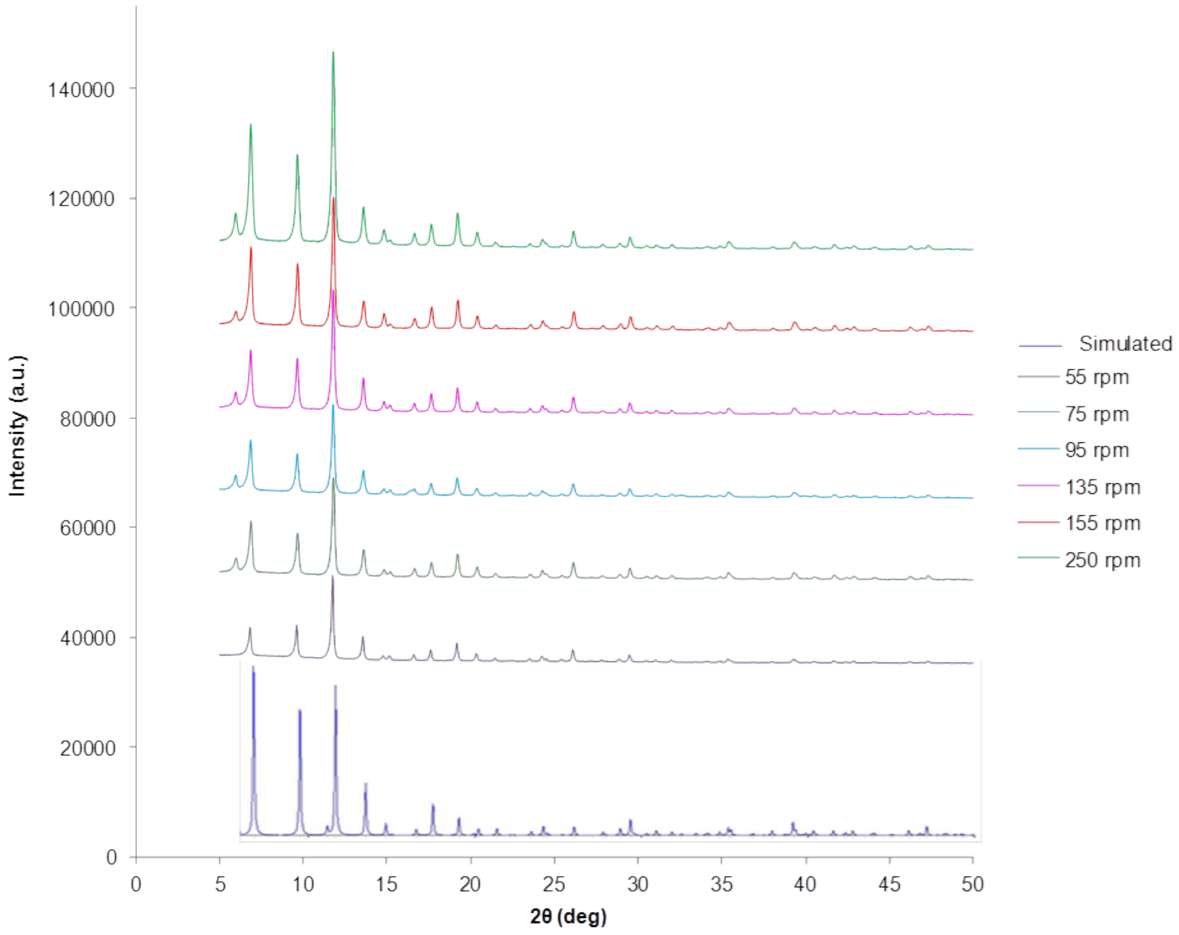

Figure 6 PXRD patterns for $Cu_3(BTC)_2$ (HKUST-1) synthesised by TSE at various of screw speeds and the simulated PXRD pattern for $Cu_3(BTC)_2$ (CSD code FIQCEN).

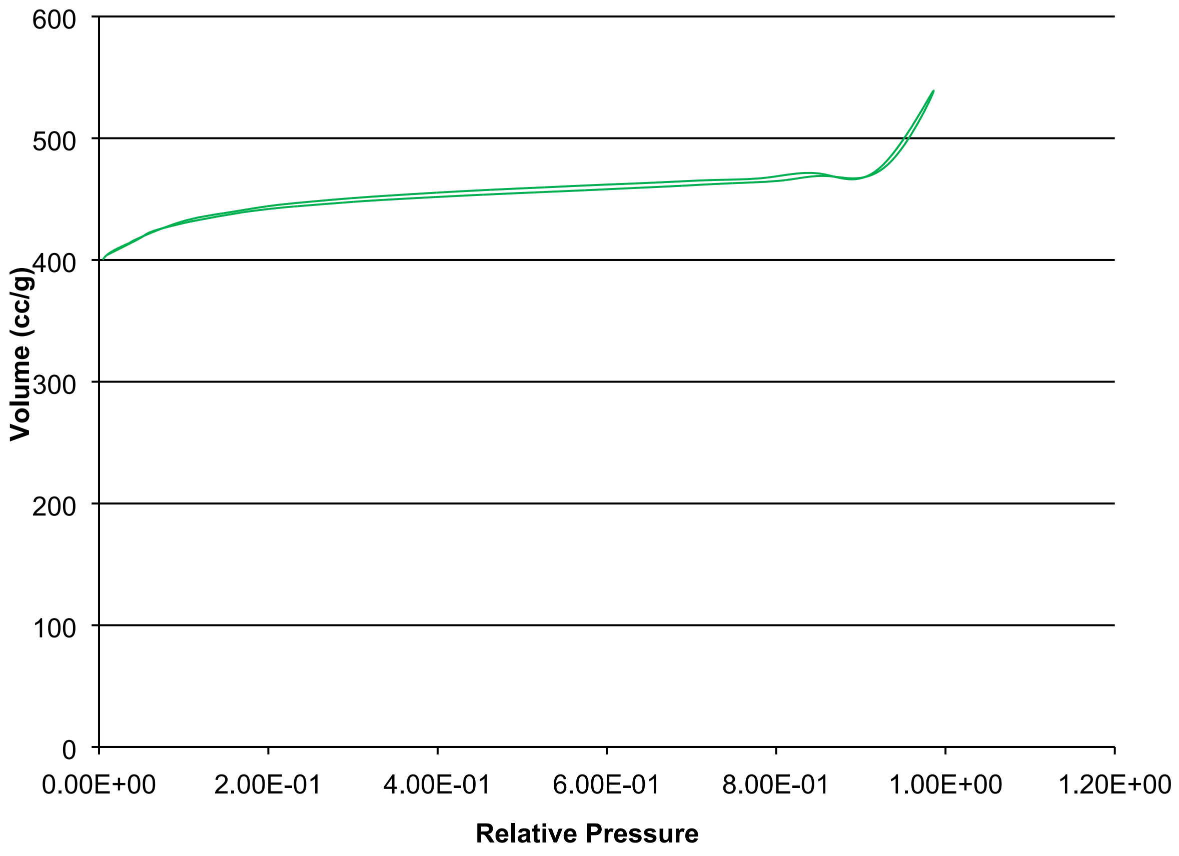

Figure 7 N_2 sorption isotherm for $Cu_3(BTC)_2$ (HKUST-1) synthesised by TSE.

Synthesis of ZIF-8

Basic zinc carbonate, $[ZnCO_3]_2 \cdot [Zn(OH)_2]_3$ (308.1 g, 0.56 moles) and 2-methylimidazole (691.8 g, 8.4 moles) were mixed by hand for 30 seconds and fed manually into a Haake Rheomex 16mm twin screw extruder, at a screw speed of 95 rpm, 75rpm or 55 rpm. The screws were configured with forward conveying sections and two kneading sections. The barrel of the extruder was heated to 200°C. A molten extrudate was produced which solidified on cooling and was collected in 5 approximately equal batches to determine homogeneity. Analysis was consistent across the five batches and PXRD patterns were very similar to that of the simulated pattern obtained from the single crystal X-ray diffraction data (CSD code FAWCEN). Activation was carried out by stirring a 5g sample in 40 mL HPLC grade methanol for 2 hours and filtering. This procedure was carried out twice. The resulting solid was oven-dried at 150°C for 2 hours. PXRD patterns for the activated products obtained at the various screw speeds were very similar to the simulated PXRD trace for ZIF-8)(Figure 8). N_2 BET Surface area: 1603.5 m^2g^{-1}; pore volume = 0.7065 cm^3g^{-1}.

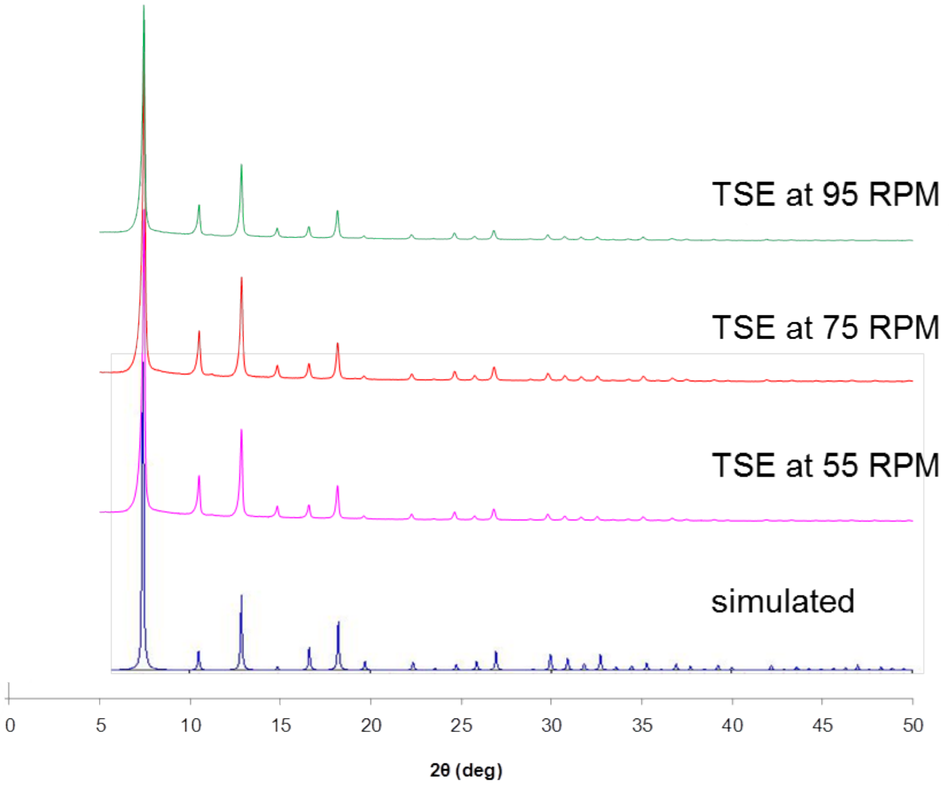

Figure 8 PXRD patterns for Zn(2-methylimidazolate)$_2$ (ZIF-8) synthesised by TSE at various of screw speeds and the simulated PXRD pattern for ZIF-8 (CSD code FAWCEN).

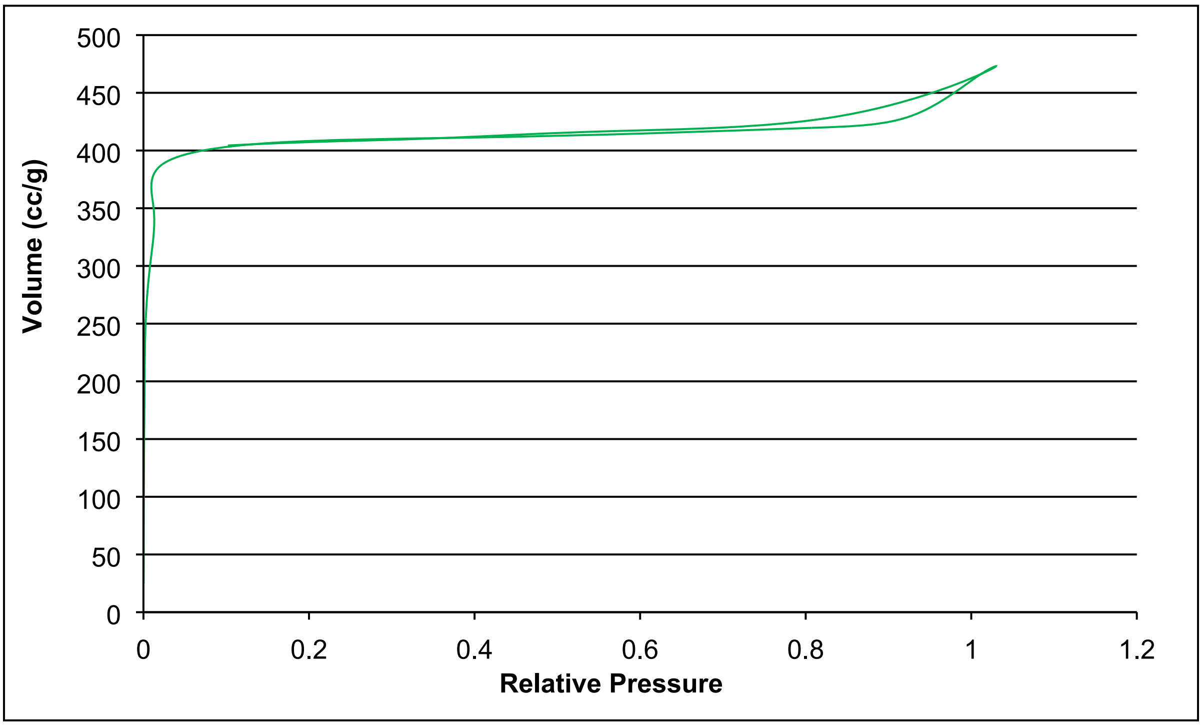

Figure 9 N_2 sorption isotherm for Zn(2-methylimidazolate)$_2$ (ZIF-8) synthesised by TSE.

Synthesis of ZIF-8 by single screw extrusion (SSE)

Basic zinc carbonate, $[ZnCO_3]_2.[Zn(OH)_2]_3$ (308.1 g, 0.56 moles) and 2-methylimidazole (691.8 g, 8.4 moles) were mixed together by hand for 30 seconds and fed at a rate of up to 70 $g.min^{-1}$ into a Dr. Collin E 25M single screw extruder with a l/d ratio of 25 at a screw speed of 30 rpm equipped with a 25 mm diameter PTFE screw of constantly increasing root diameter. The screw was configured with a conveying section and a short kneading section in the final zone. Zone 1 (feeding zone) was heated to 30^0C, zone 2 to 50^0C, zone 3 to 130^0C, zone 4 to 150 ^{o}C and zone 5 to 150 ^{o}C. The product extruded as a beige solid suspended in excess liquid 2-methylimidazole which solidified upon cooling. Several PXRD patterns of the as-synthesised extrudate were obtained to assess homogeneity and all traces were similar to the pattern simulated from single crystal X-ray diffraction data for ZIF-8 (CSD code FAWCEN), with minor deviations attributable to inclusion of 2-methylimidazole within the pores. Purification/activation was carried out by stirring a 5g sample in 40 mL HPLC grade methanol for 2 hours and collecting by filtration, twice. The solid was oven-dried at 150^0C for 2 hours. The PXRD pattern of the final product was very similar to the simulated pattern (CSD code FAWCEN) (Figure 10). N_2 BET Surface area: 1750 m^2g^{-1}; pore volume: 0.656 cm^3g^{-1}.

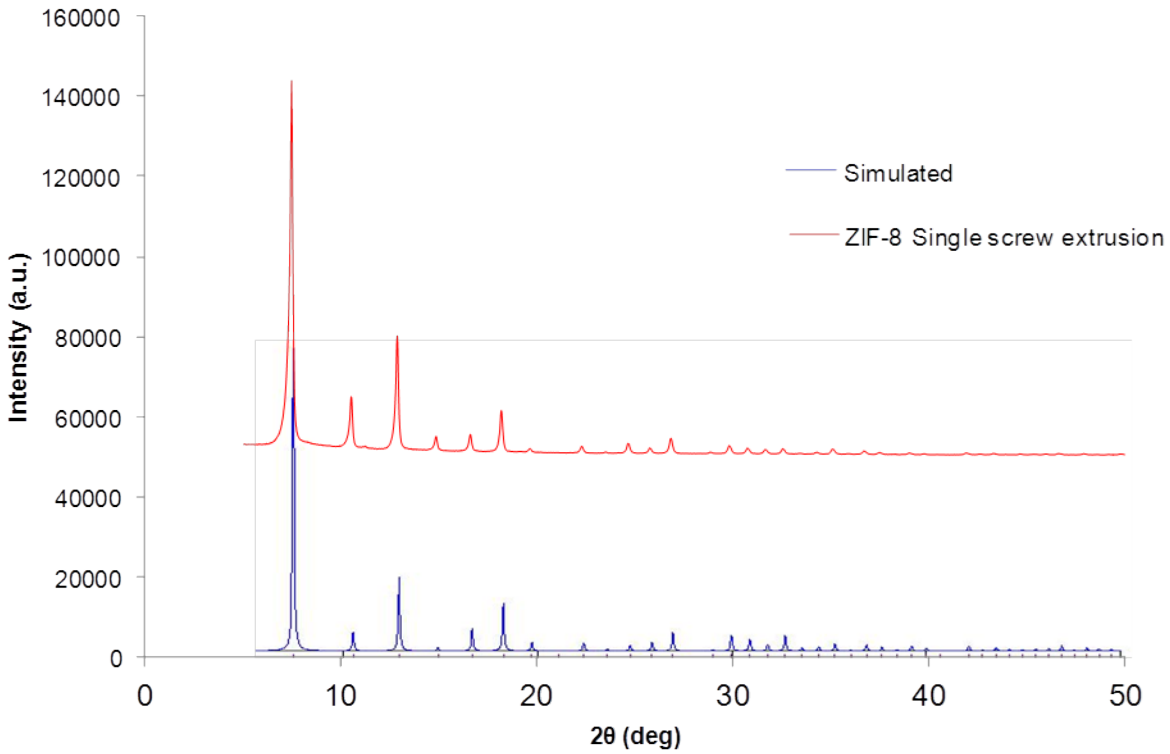

Figure 10 PXRD pattern for Zn(2-methylimidazolate)$_2$ (ZIF-8) synthesised by SSE (above) and the simulated PXRD pattern for ZIF-8 (below, CSD code FAWCEN).

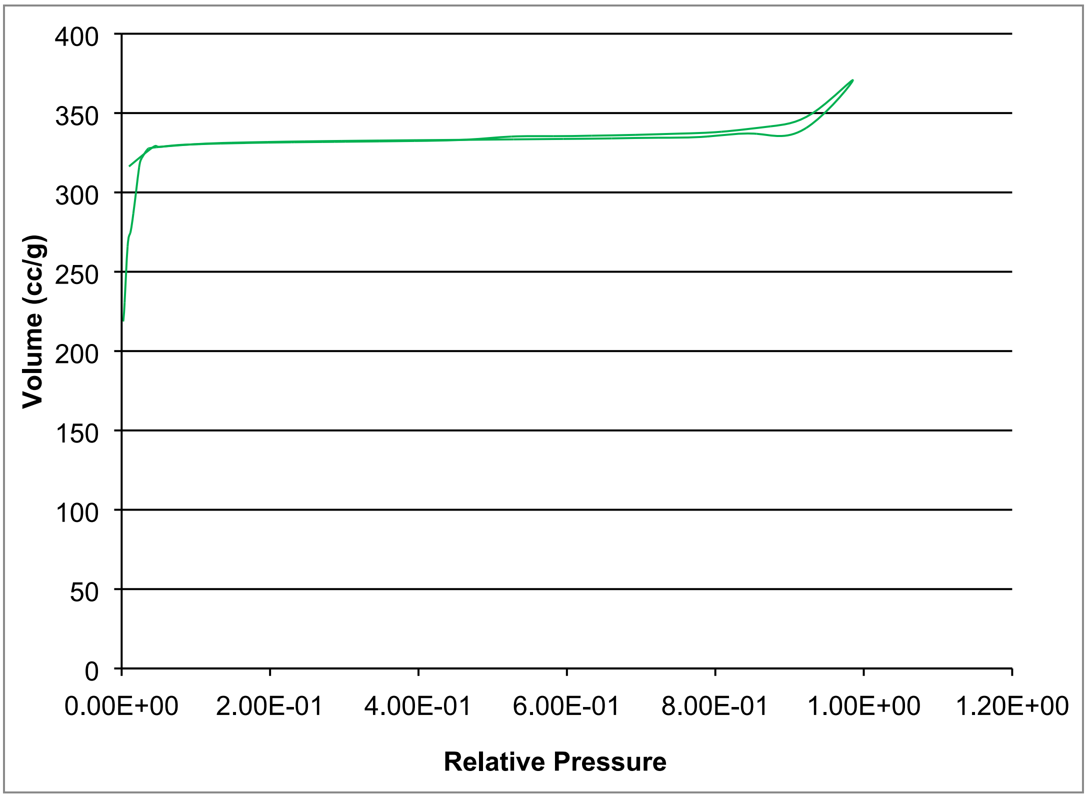

Figure 11 N_2 sorption isotherm for Zn(2-methylimidazolate)$_2$ (ZIF-8) synthesised by SSE.

Synthesis of Al(fumarate)(OH)

$Al_2(SO_4)_3 \cdot 18H_2O$ (74.48 g, 0.11 mol), fumaric acid (25.94 g, 0.22 mol) and NaOH (pellets, 26.64 g, 0.66 mol) were pre-mixed for 30 seconds and fed manually into a Haake Rheomex 16mm twin screw extruder at room temperature, a screw speed of 55 rpm and a feed rate of 10g.min^{-1}. Activation was carried out by washing 1 g of product three times with H_2O (30 ml) for 20 min. The XRD pattern of the activated product showed only the diffraction peaks corresponding to Al(OH)fumarate (Figure 12). N_2 BET surface area: 1010 m^{2}g^{-1}.

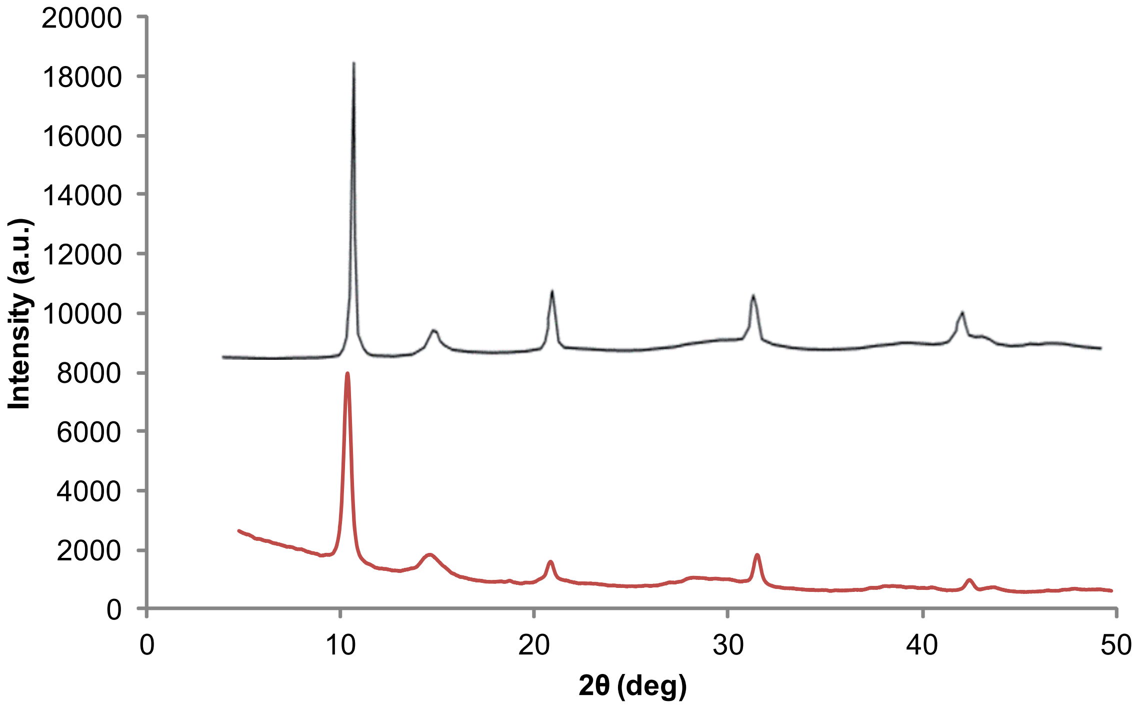

Figure 12 Comparison of the PXRD pattern for Al(fumarate)(OH) obtained by TSE (below) with that reported in the literature[1] (above).

References

1. M. Gaab, N. Trukhan, S. Maurer, R. Gummaraju, and U. Mueller, *Microporous Mesoporous Mater.* 2012, **157**, 131.
